# Supplementary figures and images for: Regulatory Role of microRNA of Milk Exosomes in Mastitis of Dairy Cows
Source: Animals (Basel). 2023 Feb 24;13(5):821. doi: 10.3390/ani13050821 (PMC10000098; doi:10.3390/ani13050821)

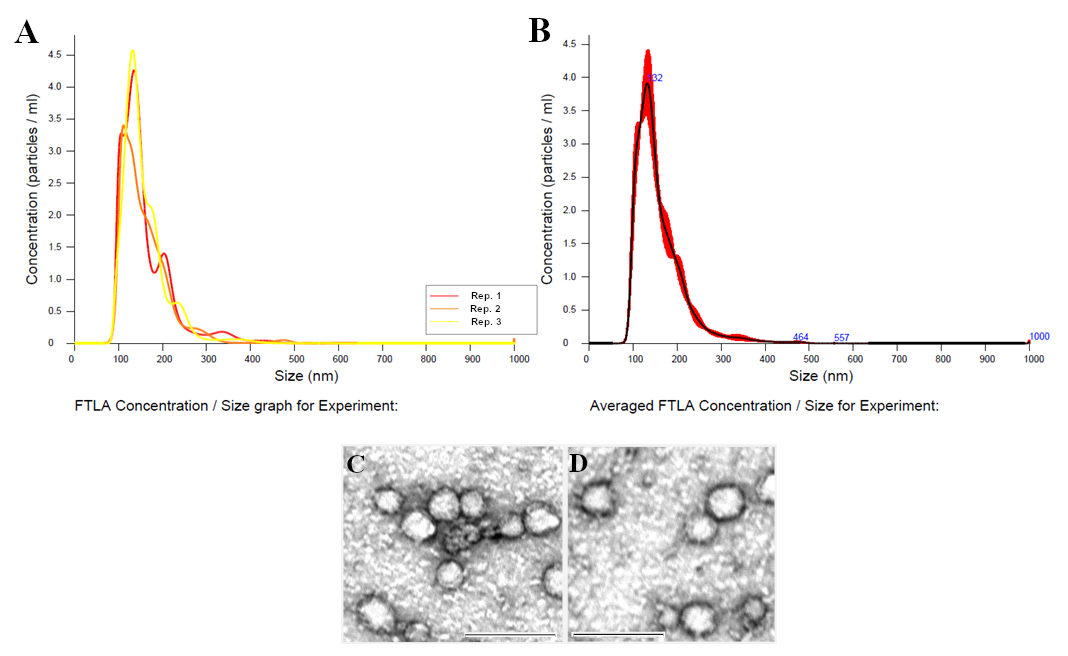

Supplement: Supplementary file 1 [file animals-13-00821-s001.zip › Suppl Figure S1.tif]
